# Bayesian-Optimized Deep Learning for *Helicobacter pylori* Detection in Egyptian Gastric Histopathology: A Multi-Architecture Study

**DOI:** 10.3390/microorganisms14102207

**Published:** 2026-10-01

**Authors:** Reham Faeq Hasona, Mahmoud M. Saafan, Eman M. El-Gendy, Walaa Ghanam, Wafaa Farouk Mohamed

**Affiliations:** 1 Computers and Control Systems Engineering Department, Faculty of Engineering, Mansoura University, Mansoura 35516, Egypt; saafan2007@mans.edu.eg (M.M.S.); eman_elgendy@mans.edu.eg (E.M.E.-G.); 2 Communications and Computers Engineering Department, Faculty of Engineering, Mansoura National University, Gamasa 35712, Egypt; 3 Pathology Department, Faculty of Medicine, Suez University, Suez 43533, Egypt; walaagg78@gmail.com; 4 Ain-Shams University Specialized Hospital, Ain-Shams University, Cairo 11588, Egypt

**Keywords:** *Helicobacter pylori*-associated gastric diseases, whole-slide images, histopathological images, Bayesian optimization, deep learning models, Vision Transformer (ViT)

## Abstract

*Helicobacterpylori* (*H. pylori*) infection is a significant health issue. It is classified by the World Health Organization as a Group I carcinogen. Egypt has unusually high prevalence rates of 64.6–90.3%, significantly above the global averages. Histopathological diagnosis is tedious and time-consuming and has high interobserver variation, especially with small, scattered bacterial colonies. This study represents the first comprehensive application of artificial intelligence to this diagnostic task. We compared eight state-of-the-art architectures. The models were trained for 50 epochs and assessed on 100 histopathological whole-slide images using metrics including accuracy, sensitivity, specificity, and AUC-ROC. MobileNetV2 and Xception performed perfectly with 100% accuracy, sensitivity, and specificity, an AUC of 1.0, and zero misclassifications. MobileNetV2 was trained 2.3 times faster, offering an efficiency advantage relevant to deployment in resource-limited pathology laboratories, pending external validation. Another four models (DenseNet-201, InceptionV3, VGG-16, and VGG-19) recorded 100% test accuracy, while Vision Transformer recorded 96.97%. These results represent retrospective patch-level classification performance and do not constitute clinical diagnostic validation. This study not only establishes the first validated artificial intelligence classification system of *H. pylori* in Egypt but also shows high performance on Egyptian samples and offers a solid methodological groundwork for future medical AI applications in resource-constrained contexts.

## 1. Introduction

*Helicobacter pylori* (*H. pylori*) is a Gram-negative bacillus that colonizes the gastric mucosa and is considered the most ubiquitous bacterial infection in the world, with nearly fifty percent of the global population infected [1]. The prevalence of the disease varies between 20% and 80% [2]. Egypt has the highest disease burden, with studies showing that *H. pylori* infection ranges between 64.6% and 90.3% for several Egyptian populations [3,4,5]: the prevalence of this disease in Egyptian school-aged children is 72.38% and as high as 90.3% in adult patients [6,7]. *H. pylori*, first identified in 1983, is a carcinogen of Group I with a relative risk of 3.8 [8]. It can be transmitted from parent to child with a high prevalence of intrafamilial transmission [9]. Among cases, 10–20% develop severe conditions such as atrophic gastritis, peptic ulcer disease, gastric adenocarcinoma, or mucosa-associated lymphoid tissue (MALT) lymphoma [4].

Histopathological examination of gastric biopsy specimens via optical microscopy with H&E and Giemsa staining is the traditional way pathologists identify *H. pylori* [10]. The small bacilli are around 2–4 micrometers in length and 0.5–1 micrometer in width [11]. Despite high sensitivity, the exceedingly small size of the bacteria in tissue sections presents a heightened risk of omission, especially when the targets are scant or distributed unevenly [12]. Mucus debris can impede the detection of bacteria [12]. The use of the Giemsa method, the high cost, and the prolonged turnaround time limit disease identification in pathology laboratories in Egypt [13]. There is evidence of moderate-to-good interobserver variability among pathologists diagnosing *H. pylori* gastritis [14], but there is a lack of gastrointestinal pathologists, particularly in rural and underresourced areas [15].

Despite the high prevalence of *H. pylori* infection in Egypt (72.4% in children and 90.3% in adults) [3,4,7], deep learning approaches remain largely absent from artificial intelligence (AI) applications in the Egyptian healthcare framework. Geographic differences in patient populations, strains of *H. pylori*, and tissue preparation and staining techniques influence the performance and generalizability of diagnostic models [16]. Such models trained on samples isolated from European or American cohorts may not perform optimally when used in Egyptian patients without thorough testing of the model [17]. *H. pylori* strains in Egypt differ in virulence gene profiles and antibiotic-resistant patterns, affecting the histopathological manifestations [6].

The latest acquisition of digital pathology and whole-slide imaging has created not only new opportunities but also new challenges on the international level, especially in Egyptian medical facilities. Despite the fact that the digitization process will increase the availability of specimens and improve consultation, it can also prove to be limited in terms of image resolution and quality of visualization [18]. It has been shown that deep learning algorithms can automate detection of *H. pylori* infection in whole-slide images [19]. Computational hardware development, particularly the use of so-called tensor processing units (TPUs) and graphical processing units (GPUs), has allowed the widespread use and optimization of deep learning model architectures to support complex medical image analysis tasks [20].

Several global studies have analyzed the use of deep learning to detect *H. pylori* with varying success. Convolutional neural networks (CNNs) achieve high accuracy in the recognition of *H. pylori* histopathologic images, though most of these studies measure only one or two architectural frameworks [16]. The bulk of the available research has relied on default hyperparameter settings or hand tuning [17], rather than advanced optimization techniques that achieve optimal hyperparameter settings [21]. The choice of hyperparameters can substantially affect model performance; nevertheless, the optimal choice of these parameters may vary greatly across different architectures and datasets [22]. Grid search is computationally infeasible, while random search lacks thorough exploration of high-dimensional hyperparameter space [23]. Bayesian optimization uses Gaussian process regression as a surrogate to estimate the dependence between hyperparameter settings and validation accuracy, to optimize the choice of future settings to evaluate, based on an expected improvement acquisition function [21,24,25].

This research aims to bridge the regional research gap with extensive and systematic deep learning architectures for the classification of *H. pylori* in Egyptian histopathological images, supplemented by Bayesian hyperparameter optimization. To our knowledge, this is the first application of deep learning for automated *H. pylori* detection in Egypt, and in general, is the first systematic evaluation of convolutional neural networks and Vision Transformer architectures for this task in the Middle East. Our contributions are as follows:

- The development of the first comprehensive implementation of AI and deep learning frameworks for classification of *H. pylori* in the context of Egyptian histopathological practices in order to provide a regional benchmark for AI-assisted diagnostic systems and establish the feasibility of local implementation.
- The use of Bayesian optimization to enable rigorous and efficient hyperparameter tuning for all the architectures used, including an extensive search space that includes the choice of optimizer (Adam, RMSprop, SGD, Adagrad, Adadelta, Adamax, or Nadam), learning rates (from 1 × 10^−2^ to 1 × 10^−5^), batch sizes (8, 16, 32, 64), and dropout configurations.
- The binary classification of histopathological images into *H. pylori*-positive and *H. pylori*-negative categories using eight different state-of-the-art deep learning architectures, including CNNs (VGG-16, VGG-19, ResNet-50, DenseNet-201, InceptionV3, Xception, and MobileNetV2) and transformer-based models (Vision Transformer) to find the best design principles for this classification.
- A comprehensive evaluation based on a multitude of performance metrics such as accuracy, precision, recall (sensitivity), specificity, F1-score, and area under the ROC curve (AUC) complemented with a detailed analysis of the diagonal confusion matrix.
- An analysis of the efficiency of training and computational requirements in order to inform practical deployment strategies in resource-constrained clinical environments that are characteristic of Egyptian pathology laboratories.

Section 2 summarizes previous deep learning approaches to *H. pylori* identification. Section 3 presents deep learning, convolutional neural networks (CNN), transfer learning, hyperparameter optimizer, and Bayesian Optimizer. Section 4 describes our dataset implementation. Section 5 reports performance across training, validation, and testing. Section 6 discusses the clinical relevance of perfect classification accuracy and future directions. Section 7 concludes with key insights and recommendations.

## 2. Related Works

Deep learning approaches for *H. pylori* detection in histopathology have evolved from single CNN classifiers to more sophisticated pipelines. Early ensemble and patch-based CNN models demonstrated strong diagnostic performance when validated against pathologist assessment [18,26], while comparative studies against traditional microscopy and immunohistochemistry showed that deep learning can achieve higher sensitivity, positioning it as a potential screening aid rather than a replacement for expert diagnosis [10,27]. The development of large, curated datasets such as DeepHP [20] enabled systematic benchmarking of standard architectures (VGG16, ResNet50, InceptionV3), while subsequent work explored five-fold cross-validation on smaller datasets [16] and hybrid CNN–gradient-boosting architectures [28] to improve robustness.

More recent studies have addressed clinical translation more directly: two-stage models combining classification with lesion localization [29] and systematic comparisons of CNNs against AutoML pipelines that also tested external/out-of-distribution validation, revealing substantial performance drops (from 97% to 78% accuracy) when models were applied to unfamiliar staining and imaging conditions [17]. This generalizability challenge, together with the reliance on relatively small single-institution datasets across the literature, motivates systematic architecture comparison and rigorous hyperparameter optimization undertaken in the present study.

## 3. Background

This section presents the theoretical background of the techniques employed in this study.

### 3.1. Deep Learning

Deep learning is a branch of machine learning that uses algorithms to model data in terms of a series of multilayered nonlinear transformation functions. Deep learning has a powerful architecture, which allows the model to better represent data in terms of layers added. To overcome the problem of pattern extraction from large datasets, deep neural networks are essential, thereby making computers learnfaster and more accurately. This model is known as deep learning and is used more frequently both in scientific studies and in practice [30].

Our eight deep learning frameworks in this study include seven pretrained models (MobileNetV2, DenseNet201, Xception, Inception, ResNet50, VGG16, and VGG19) that are based on CNNs and one model that is based on a Vision Transformer. These are the most common deep learning models (see Table 1).

### 3.2. Convolutional Neural Networks (CNNs)

Convolutional neural networks (CNNs) are a branch of sophisticated learning techniques that are used in processing visual data [31]. CNNs are typically characterized by three types of layers, namely, the convolutional layer, the pooling layer, and the fully connected layer. The first two layers (i.e., the convolutional and pooling layers) are used in order to extract features. The terminal layer (i.e., the fully connected layer), in its turn, matches the extracted objects to the final output space [32].

### 3.3. Transfer Learning

Transfer learning is a model that involves the use of a pretrained model on a dataset to solve other relevant problems. Implementation of transfer learning is done using the pretrained model as a starting point that will be altered in parameters so that it can fit in the new dataset [33]. As a result, transfer learning represents a method with a simple idea, which consists of utilizing the knowledge gained within certain models and transferring it to the necessary tasks and fields. There are multiple pretrained CNN models that have been trained on the ImageNet image dataset [34], but the models used in the present study include MobileNetV2, DenseNet201, Xception, Inception, ResNet50, VGG16, and VGG19.

### 3.4. Hyperparameter Optimizer

Current modeling methods often involve various parameters in the data training process. In contrast to other parameters that can change during the training process, there are immutable parameters known as hyperparameters. Hyperparameters are important to define as they can affect the reliability and performance of modeling techniques. Hyperparameters are parameters that are used to adjust and control the modeling method to produce better prediction performance [34,35].

### 3.5. Bayesian Optimizer

Deep learning optimization includes a black-box optimization problem with the objective function f(y). It is important to reduce the number of samples over multiple steps across layers. Bayesian optimization is very useful in this domain, where human expertise cannot provide better accuracy. Bayesian optimization includes prior information about the function f and updates the posterior information, which helps reduce losses and maximize model accuracy [25].

**Table 1:** Deep learning models summary.

Model	Type	Year	Depth	Parameters	Key Innovation	Primary Strength	Main Limitation
MobileNetV2 [36]	CNN	2019	Variable	4.2M	Depth-wise separable convolutions + NAS	Extreme efficiency	Lower capacity for complex tasks
DenseNet201 [37]	CNN	2017	201	18.3M	Dense connectivity	Feature reuse, gradient flow	Memory-intensive during training
ResNet-50 [38]	CNN	2016	50	23.6M	Residual convolutions	Enables very deep networks	Can overfit on small datasets
InceptionV3 [39]	CNN	2016	48	21.8M	Multi-scale parallel convolutions	Captures features at multiple scales	Complex architecture
Xception [40]	CNN	2017	71	20.9M	Extreme depth-wise separable convolutions	Efficient spatial-channel decoupling	Requires careful tuning
VGG-16 [41]	CNN	2014	16	14.7M	Simple uniform 3 × 3 convolutions	Simplicity, interpretability	Computationally expensive
VGG-19 [41]	CNN	2014	19	20.0M	Deeper uniform 3 × 3 convolutions	Slightly better than VGG-16	Even more computationally expensive
Vision Transformer (ViT) [42]	Transformer	2020	12 layers	85.8M	Pure attention mechanism	Long-range dependencies, scalability	Requires large datasets, slow inference

## 4. Materials and Methods

### 4.1. Dataset

The images used to build the dataset were collected from gastric biopsies stained with Giemsa and regular hematoxylin-eosin (H&E) using histopathological whole-slide imaging (WSI) of 100 gastric biopsies from 50 patients (68 *H. pylori*-positive and 32 *H. pylori*-negative biopsies) taken by one pathologist. All digital images were taken at 40× magnification using an Olympus CX23 Binocular microscope (Evident, Tokyo, Japan), with a 0.5× magnification relay lens, connected to an MC500-G3 USB Microscope Digital Camera (Lanoptik Technologies Ltd., Guangzhou, China), 5.0 megapixel. Other parameters were as follows: camera pixel size, 2.2 μm × 2.2 μm (BSI); image size, 2592 × 1944 pixels; image format, * .png; pixel bit depth, 24 bits (RGB three-channel image, 8 bits per channel, 3 × 8 = 24). Histopathological samples are anonymous images of human gastric mucosa embedded in paraffin, fixed in formalin, and stained with Giemsa and regular H&E from the retrograde study of 100 cases of gastric endoscopic biopsies, obtained from the archives of the Pathology Department of Kafer El Sheik General Hospital. The clinical data, which encompassed the patient’s age and sex, were gathered from the pathology records and patients’ medical files.

### 4.2. Image Preprocessing

**Figure 1:**
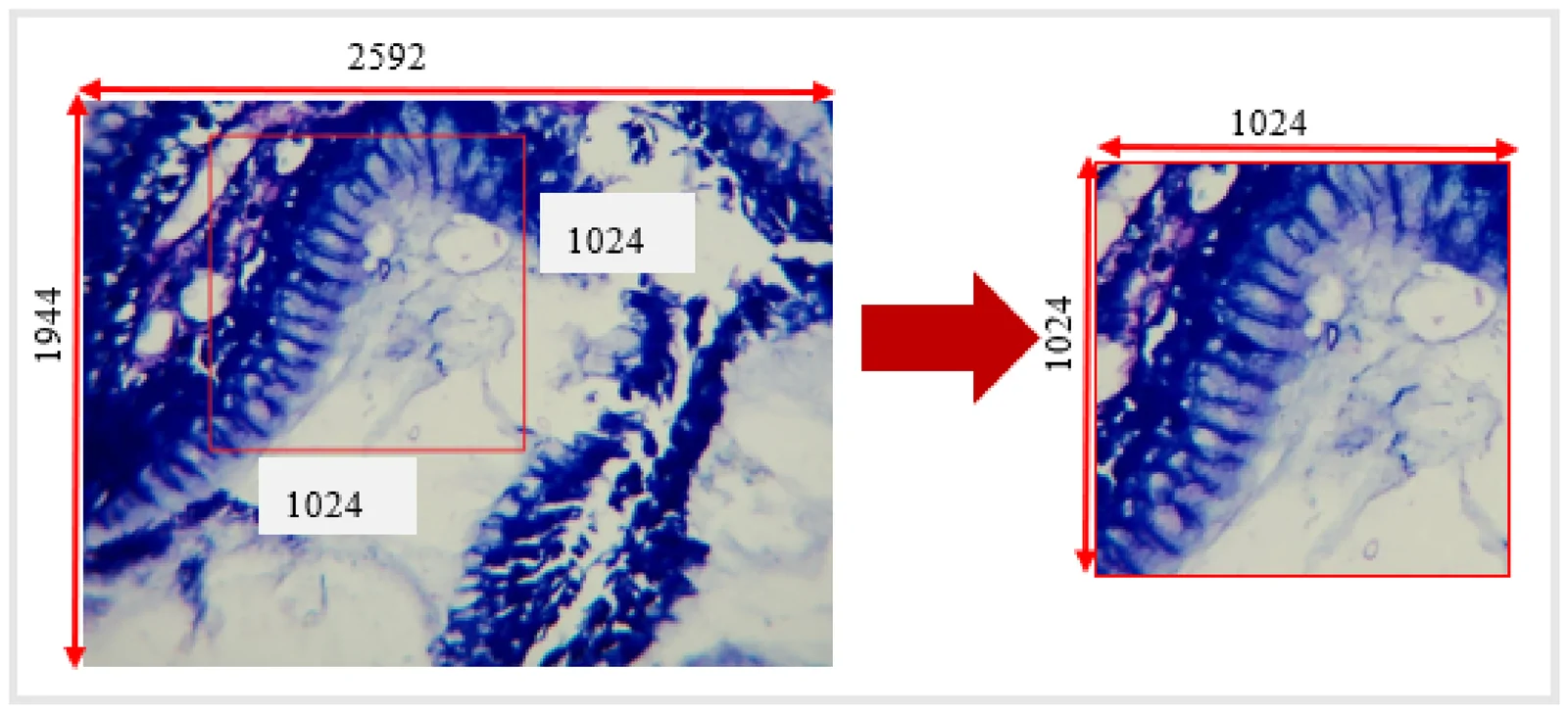
A random example of an original WSI (2592 × 1944 pixels) and one patch (1024 × 1024 pixels).

In order to maximize the use of the high-resolution whole-slide images (WSI) and produce a suitable number of training samples from the existing gastric biopsies, the original WSI images (2592 × 1944 pixels) were systematically partitioned into smaller, non-overlapping image patches of 1024 × 1024 pixels through the implementation of grid sampling techniques (see a random sample in Figure 1). This grid-based sampling methodology guaranteed comprehensive coverage of each whole-slide image without redundancy, as patches were systematically extracted, ensuring no overlap occurred between adjacent patches.

In the patch extraction procedure used on the collection of whole-slide images, 432 image patches of 1024 × 1024 pixels were generated. Ground truth at the patch level was established through a targeted, pathologist-guided procedure rather than a uniform inheritance of the parent slide’s diagnostic label. Within each *H. pylori*-positive whole-slide image, regions containing visually identifiable bacilli were first precisely localized; positive patches were then extracted exclusively from these confirmed bacteria-containing regions, rather than from the slide in its entirety. In contrast, negative patches were extracted from *H. pylori*-negative slides across the full tissue area, reflecting the confirmed absence of the organism. Following extraction, all patches (both positive and negative) underwent a review step to verify the accuracy of the assigned label prior to inclusion in the final dataset. This procedure was adopted specifically to prevent the label noise that would otherwise result from the sparse, nonuniform distribution of *H. pylori* within infected gastric mucosa, ensuring that each patch label accurately reflects the histopathological content of that specific image region rather than the diagnosis of the parent slide as a whole. This patch-based model is widely used in digital pathology to allow deep learning models to learn high-resolution tissue features and deal with computational constraints.

All 432 patches were preprocessed in a standardized way for modeling after they were extracted. To lessen computational requirements while keeping key morphological features for classification, each 1024 × 1024-pixel patch was resized down to 512 × 512 pixels. We selected this resolution to try to balance preserving fine-scale patch architecture with the constraints on GPU memory capacity and training efficiency. The 512 × 512 resolution contains enough detail to identify the characteristic spirally or curved rod morphology of *H. pylori* with a length of around 2–4 micrometers, while allowing batch processing while training the model.

Prior to patch extraction, whole-slide images were assigned to two mutually exclusive patient cohorts according to *H. pylori* status, with no patient represented in both the positive and negative groups. The 50 patients were partitioned into 30 for training (*H. pylori*-positive, 10 negative), 10 for validation (seven positive, three negative), and 10 for testing (seven positive, three negative). Following this patient-level assignment and patch extraction, all 1024 × 1024 patches originating from a given patient’s slide(s) were kept together and assigned in their entirety to a single subset (training (~70%), validation (~15%), or test (~15%)) (as shown in Table 2) such that no patch from a given patient appeared in more than one subset. This patient-level splitting strategy was adopted specifically to eliminate the risk of data leakage arising from shared staining characteristics, tissue morphology, or imaging artifacts among patches drawn from the same patient, thereby ensuring that the reported test-set metrics reflect genuine generalization to entirely unseen patients rather than memorization of patient-specific features. The stratification further preserved the proportional representation of H&E and Giemsa-stained material, as well as class balance, across all three subsets.

### 4.3. Data Augmentation

Data augmentation [43] was applied exclusively to the training set, following the patient-level stratified splitting; the validation and test subsets contained only original, non-augmented patches throughout model training, hyperparameter optimization, and evaluation, thereby precluding any leakage of augmented test-image derivatives into the training process.

The training set comprised 300 patches with a perfectly balanced class distribution (150 positive and 150 negative). However, to enhance model generalization and prevent overfitting given the modest size of the training set, on-the-fly data augmentation was applied exclusively to the training set using the Keras ImageDataGenerator. This dynamic augmentation technique applies random transformations (such as rotation, shifting in width and height, shearing, zooming, and horizontal and vertical flipping, as listed in Table 2) to each image during each training epoch, generating a diverse set of transformed versions without creating a fixed, physically stored set of augmented images. This approach effectively exposes the model to a virtually infinite variety of training samples, improving its ability to generalize to unseen data. The validation and test sets (66 patches each) were not augmented to preserve the integrity of unbiased performance evaluation [44,45,46].

Data augmentation makes it easier for the model to generalize to previously unseen data. This is especially useful when the original dataset is small or when the images come from resource-limited settings where there is a lot of variability. By generating multiple variations of each image, the model becomes less likely to overfit on the training images and learns more generalized patterns, which ultimately help with the classification of new images.

### 4.4. Bayesian Optimization for Hyperparameter Tuning

We used Bayesian optimization implemented in Keras Tuner as a principled and efficient approach for the search of optimal training configurations for each architecture due to the large hyperparameter search space and the cost of training deep neural networks, especially in evaluating multiple architectures with different optimal configurations. Bayesian optimization is a sequential model-based optimization technique. It builds a probabilistic surrogate model of the objective function (which could be taken as validation accuracy). So, we can maximize the hyperparameter configurations using an effective tradeoff between exploration and exploitation. In other words, we can balance the exploration of regions with uncertain predictions and the exploitation of regions with high predicted performance. The hyperparameter space was fully explored, including all important training parameters (see Table 3).

The surrogate model used by the Bayesian optimization algorithm to predict validation accuracy from the hyperparameters is a Gaussian process regression. The Gaussian process gives us not only the mean performance we expect given any particular hyperparameter configuration, but also an uncertainty measure. This is essential to balance exploration versus exploitation. The next hyperparameter combinations to evaluate were selected based on an expected improvement acquisition function, which balances the exploration of undersampled regions (where uncertainty is high) against the exploitation of promising areas in hyperparameter space (where predicted performance is high).

For each model architecture, the optimization process involved iteratively evaluating 50–80 different hyperparameter configurations. This fixed 10-epoch training window per trial was configured within a tuner setup permitting a maximum budget of 100 epochs, with early stopping (patience of 10 epochs) and learning rate reduction (patience of 5 epochs) available as safeguards; in practice, the 10-epoch window was sufficient to obtain a stable comparative estimate of validation performance across trials without triggering these thresholds. Following selection of the optimal configuration per architecture, final models were retrained under a separate 50-epoch budget with correspondingly scaled early stopping (patience of 5 epochs) and learning rate reduction (patience of approximately 2–3 epochs), consistent with the convergence epochs. With every iteration, the selection of the next configuration was guided by what was learned previously in the performance landscape. While the main goal of optimizing the model was validation accuracy, the second objective was training time per epoch. The latter helped in locating configurations that were not only achieving good validation accuracy but also not too costly to compute. This was necessary so that we could deploy these models in practice.

### 4.5. Performance Metrics

Model selection involved a detailed assessment using traditional performance measures: accuracy, area under the ROC curve (AUC), precision, recall (i.e., sensitivity), F1 score, and specificity, as well as error distribution using confusion matrices. As per effective classification, TP—true positive, TN—true negative, FP—false positive, and FN—false negative refer to cases correctly classified as a positive case, correctly classified as a negative case, misclassified as a positive case, and misclassified as a negative case, respectively. Table 4 presents the performance metrics that were used in the study.

## 5. Experimental and Result

The purpose of this study was to explore how the optimizer, learning rate, batch size, and dropout configuration influenced the ability of the model to classify *H. pylori* images effectively, as well as to select the best optimized hyperparameters and models.

Transfer learning was implemented as a frozen backbone feature extraction across all eight architectures evaluated in this study: the ImageNet-pretrained convolutional base of each model was kept entirely nontrainable, while a newly appended classification head comprising global average pooling, two dense layers with dropout (searched as a hyperparameter, Table 3), and a final softmax output layer was trained on the *H. pylori* classification task. This frozen backbone approach, applied uniformly across all models, was adopted given the modest training set, where full fine-tuning risked overfitting due to the large number of backbone parameters relative to available training examples. Input images were normalized via min-max rescaling to the [0, 1] range (rescale = 1/255) rather than each architecture’s specific ImageNet preprocessing function; we note this as a simplification applied consistently across all architectures relative to standard ImageNet preprocessing pipelines, in which architecture-specific normalization could potentially refine further in future work.

All the experiments were conducted using an NVIDIA Tesla T4 GPU, which was available on Google Colaboratory (Colab) (online), a dependable collaborative platform for image analysis based on a Jupyter/iPython notebook-configurable environment. The Keras (version 3.13.2) framework was used in developing all the deep learning models with TensorFlow (version 2.20.0) as the Keras backend. Google Colab has provided the following cloud workstation specifications: dual-core Intel(R) Xeon(R) CPU @ 2.20 GHz, 12.7 GB RAM, and NVIDIA Tesla T4 16 GB GPU. Table 5 illustrates the experimental arrangement used for each model.

### 5.1. Bayesian Optimization Outcomes

The process of Bayesian optimization was able to discover optimal hyperparameter configurations for each evaluated deep learning architecture based on maximizing validation accuracy (score = 0.98 to 1) in this study. The ideal hyperparameter search space for each of the models is listed in Table 6. Many relevant configurations across architectures emerged from the optimization, as well as architecture-specific choices that represented the different training dynamics and regularization demands of each model.

### 5.2. Performance Across Training, Validation, and Test Sets

After training for 50 epochs with the optimal hyperparameters, we start from the most important distinction between validation and test set performance; that is, good performance on the test set indicates better generalization ability for unseen samples that were not used during hyperparameter optimization. The training time per epoch was monitored as a secondary consideration, in order to identify configurations where better performance was achieved without high training cost, which will be important for practical deployment feasibility. The information gleaned from the experiments is presented in Table 7. The results provided us with useful insight into the performances of the models in various configurations, which helped us optimize the architecture and optimization choices. Table 7. The best configuration of hyperparameters for all models.

**Figure 2:**
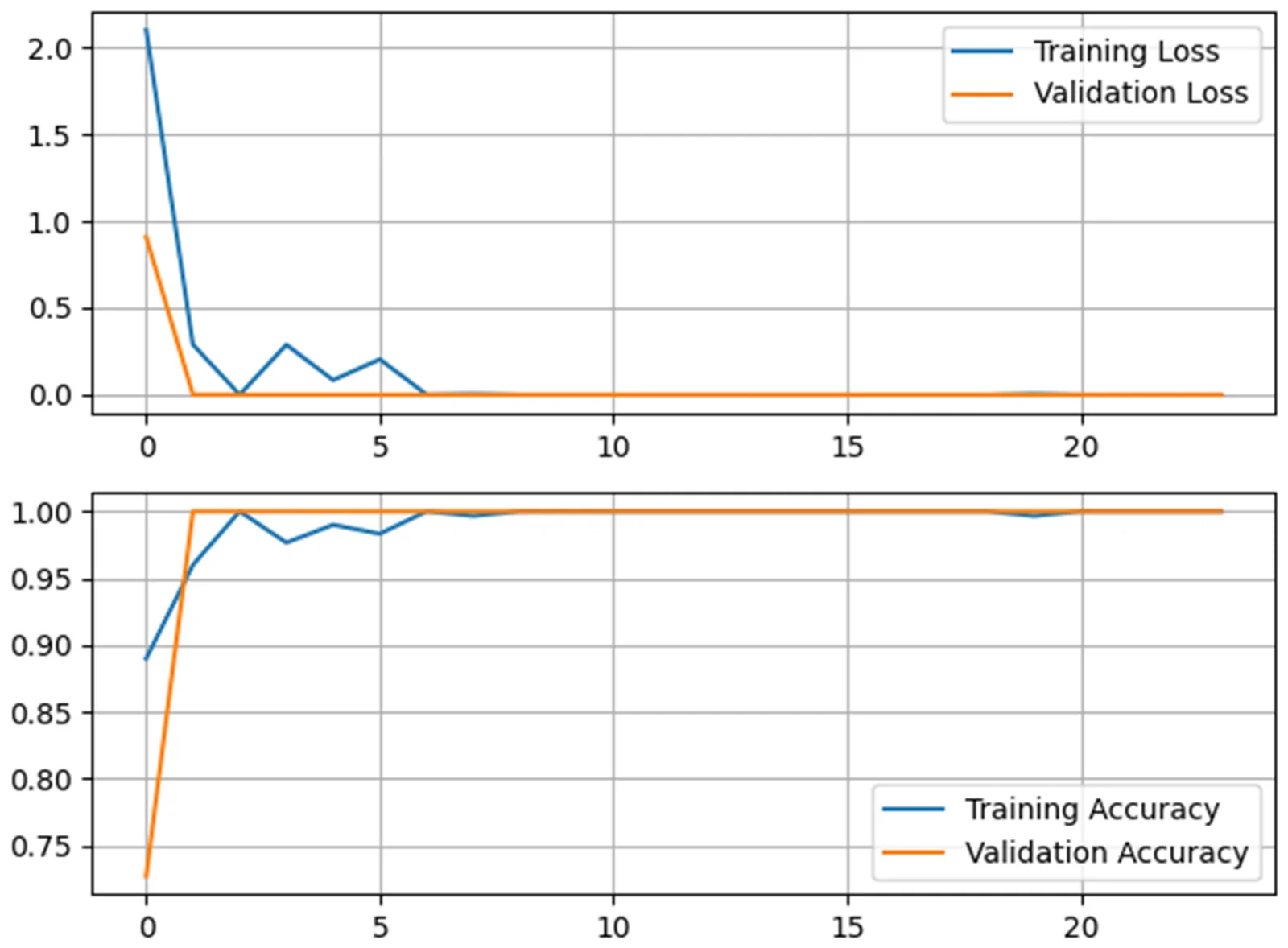
Training and validation loss and accuracy curves of MobileNetV2.

**Figure 3:**
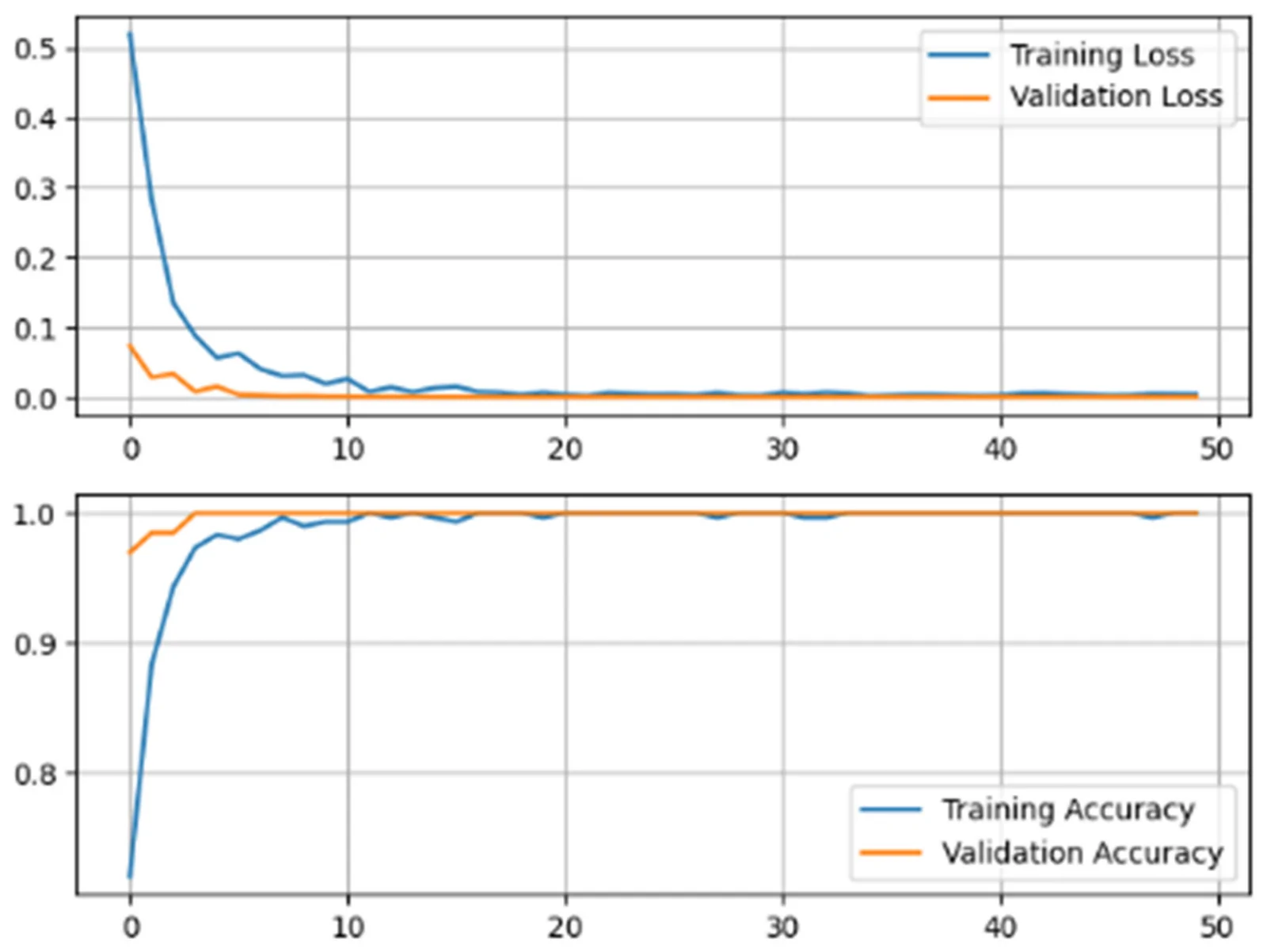
Training and validation loss and accuracy curves of DenseNet201.

**Figure 4:**
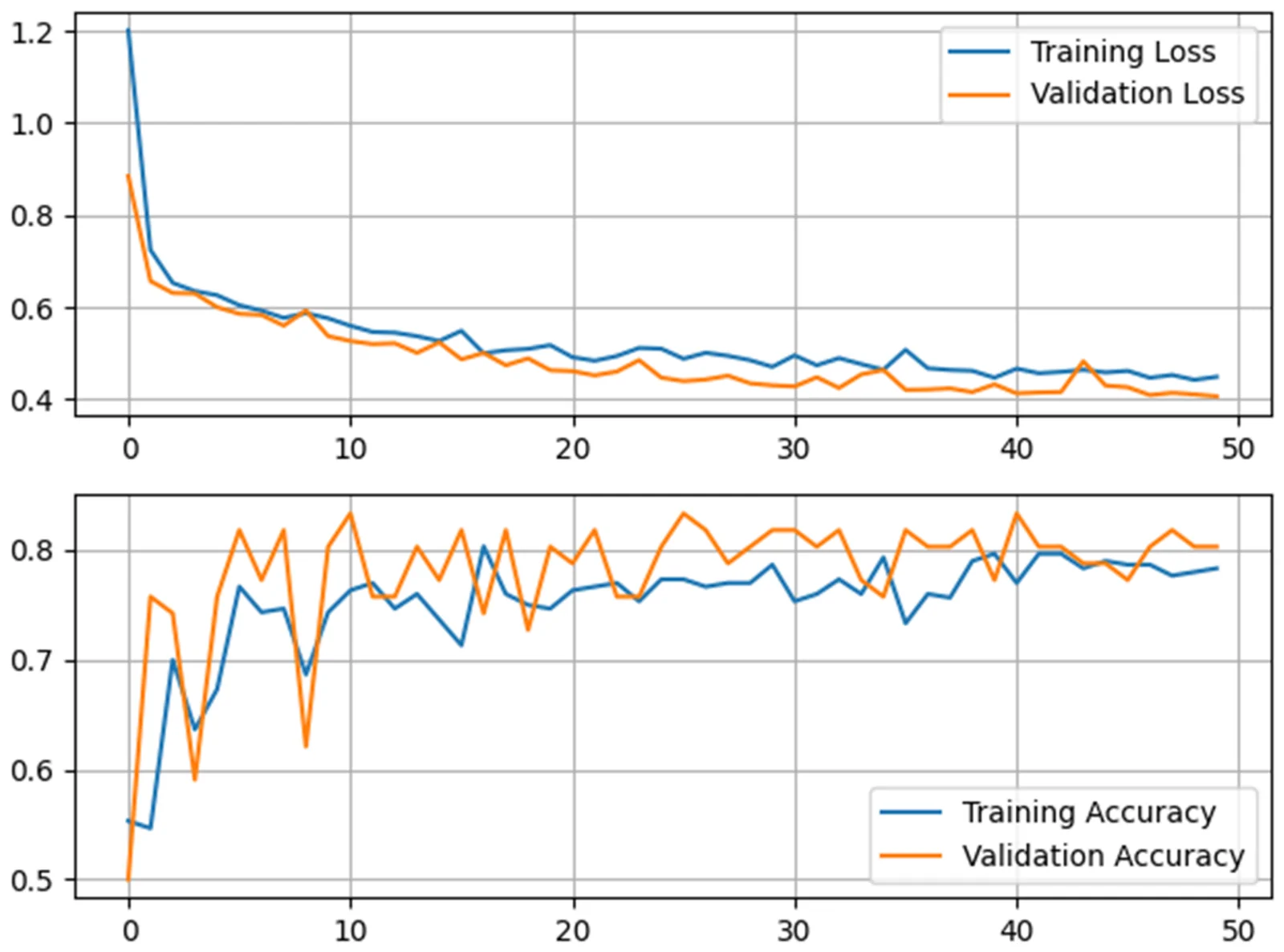
Training and validation loss and accuracy curves of ResNet50.

**Figure 5:**
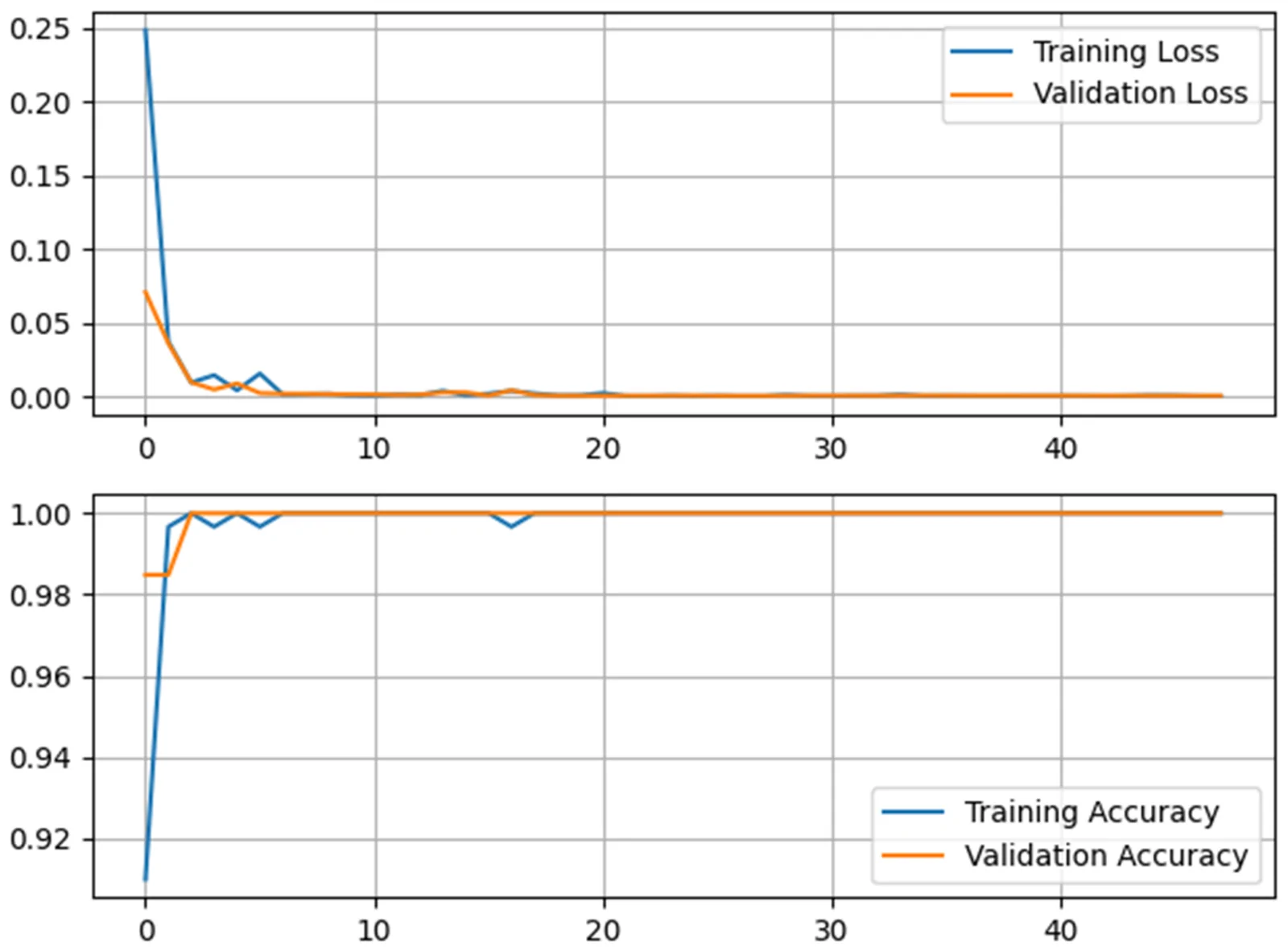
Training and validation loss and accuracy curves of InceptionV3.

**Figure 6:**
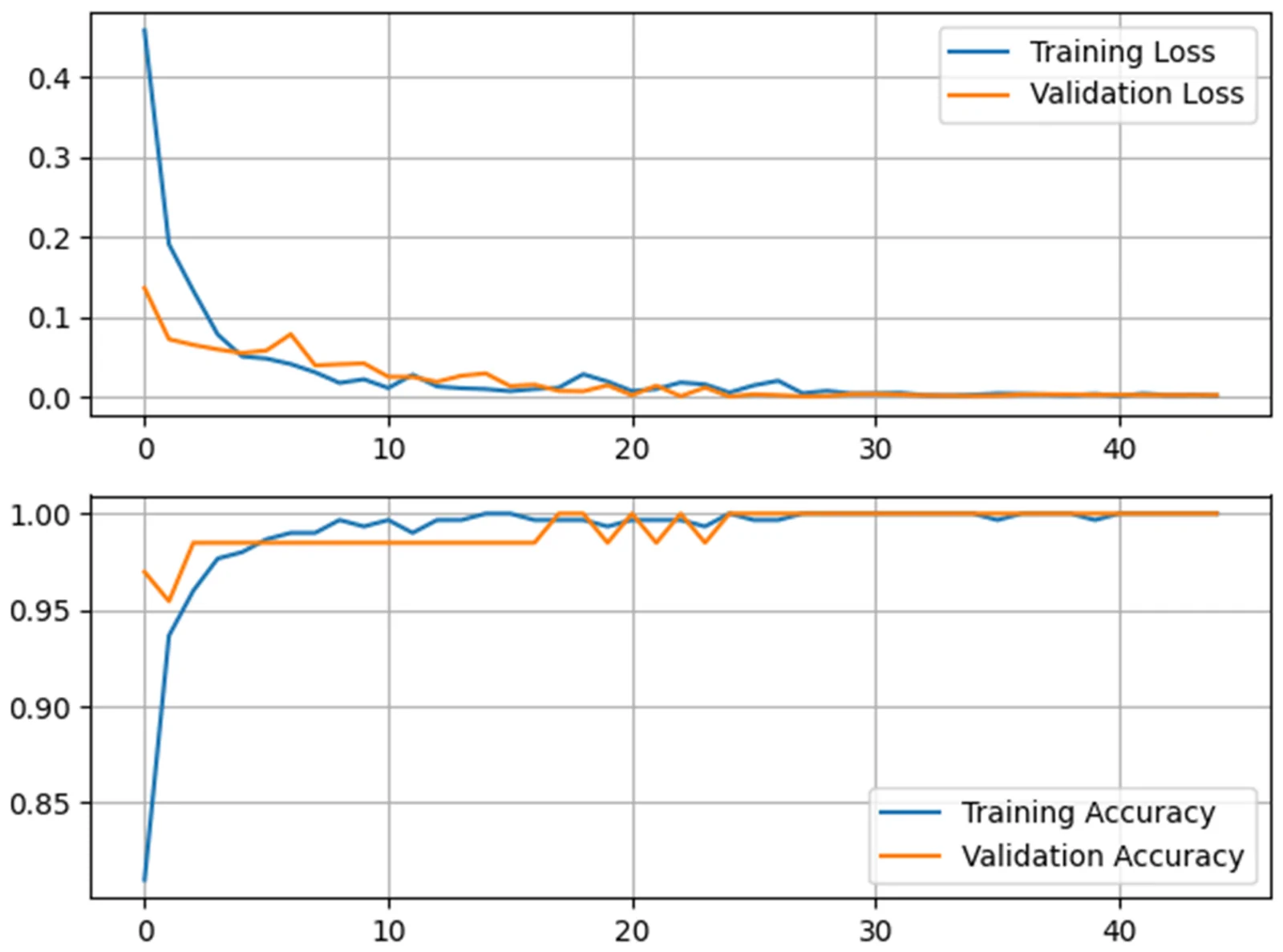
Training and validation loss and accuracy curves of Xception.

**Figure 7:**
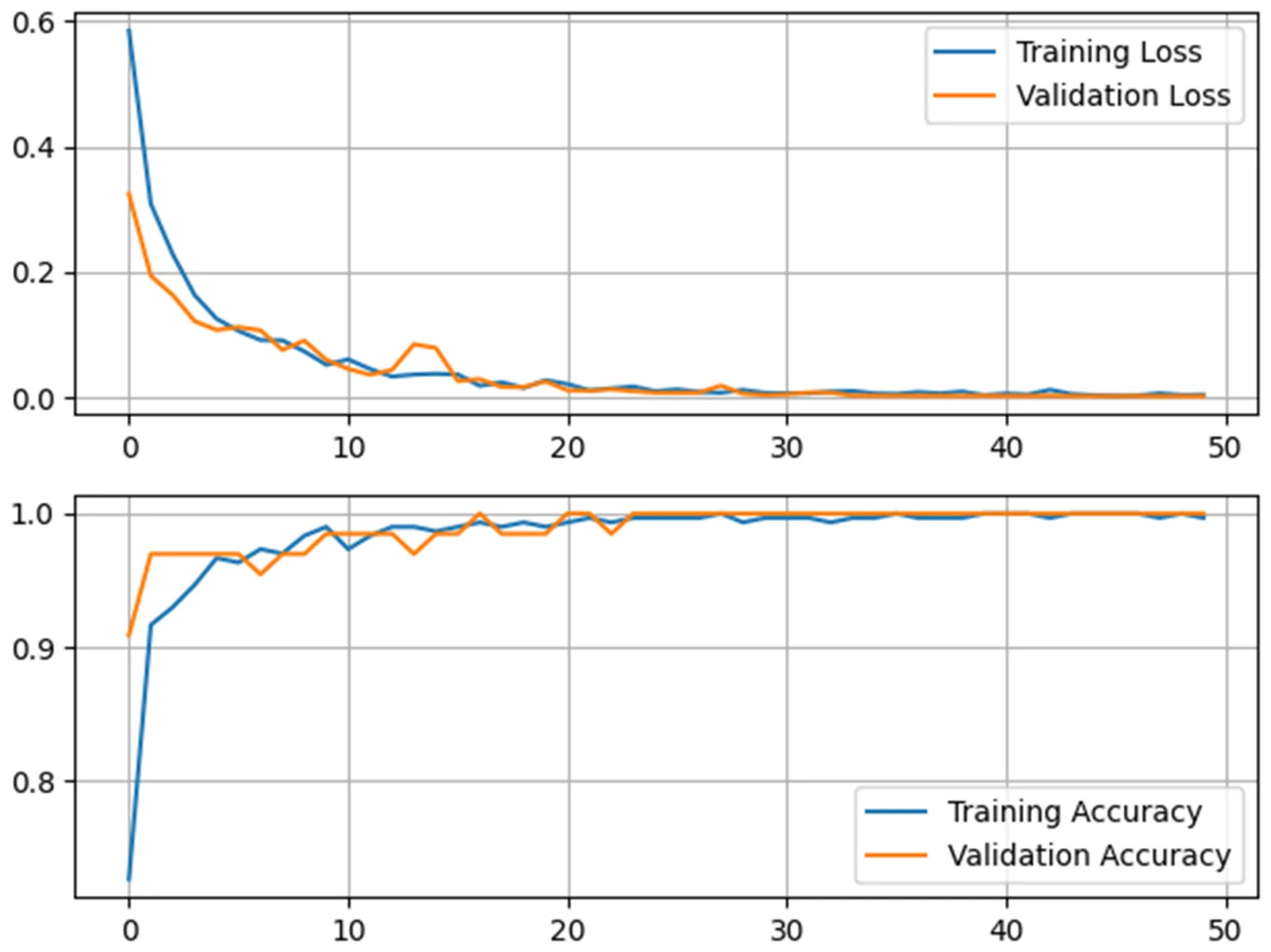
Training and validation loss and accuracy curves of VGG16.

**Figure 8:**
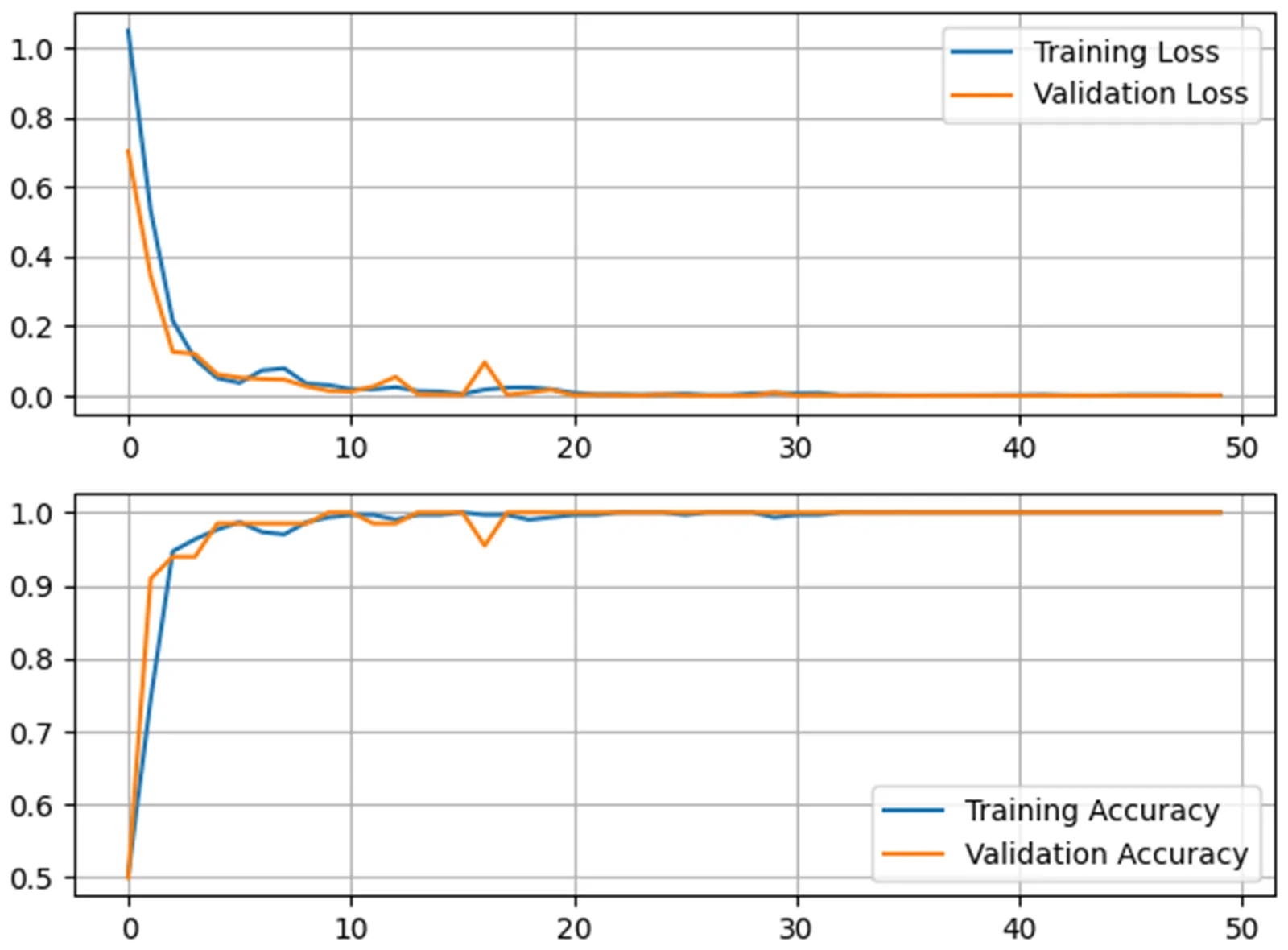
Training and validation loss and accuracy curves of VGG19.

**Figure 9:**
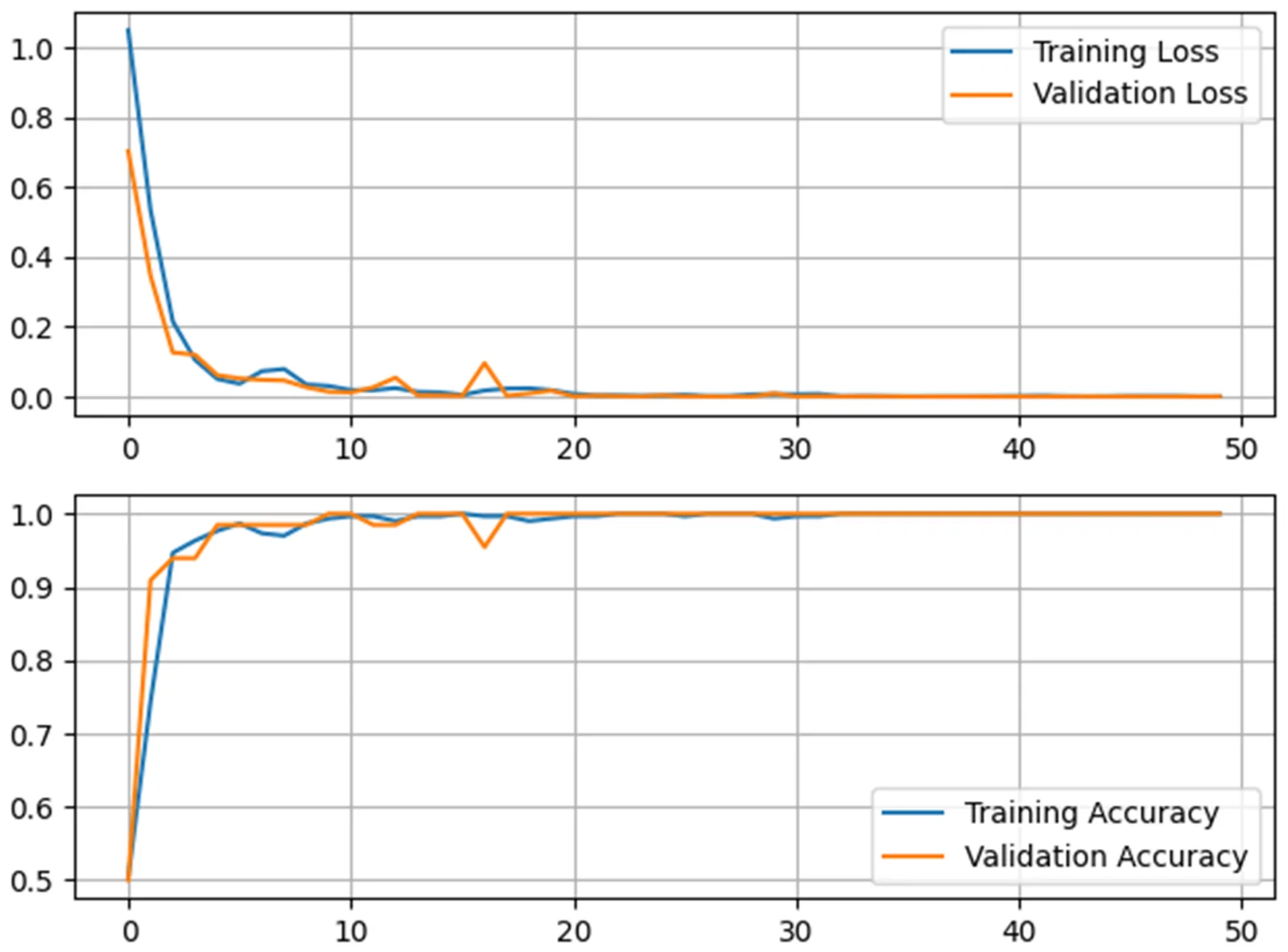
Training and validation loss and accuracy curves of ViT.

**Figure 10:**
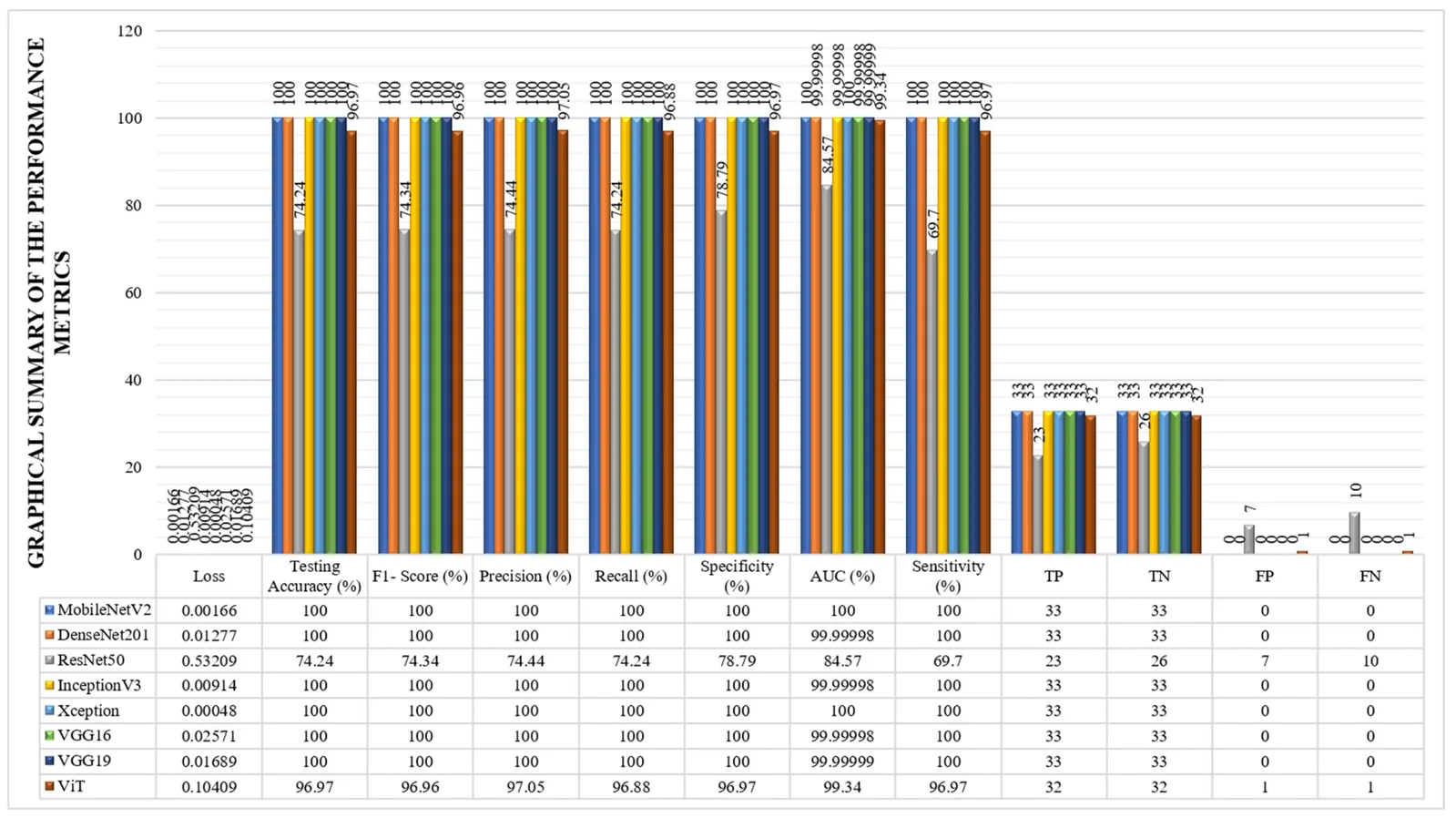
Graphical summary of the performance metrics for each model. It indicates that MobileNetV2 and Xception are the best models.

From Table 7, the best configuration of hyperparameters for each model was identified as having the highest accuracy and the lowest training time per epoch to achieve the best performance for classifying *H. pylori*, as shown in Table 8, which now also reports the corresponding 95% confidence intervals (Clopper-Pearson exact method) for testing accuracy. Also, see Figure 2, Figure 3, Figure 4, Figure 5, Figure 6, Figure 7, Figure 8 and Figure 9 for further clarification on the relationship between training accuracy and validation accuracy and also the loss between training and validation. It is clear that the Adam parameter optimizer is the best choice for three models, and the RMSprop as well as the Nadam parameter optimizers are the best choices for two models each. A learning rate of 0.001 is the best choice across five models. A batch size of 32 is the best choice using six models. Figure 10 shows the relevant performance metrics for these best configurations following the learning and optimization processes for each model.

**Figure 11:**
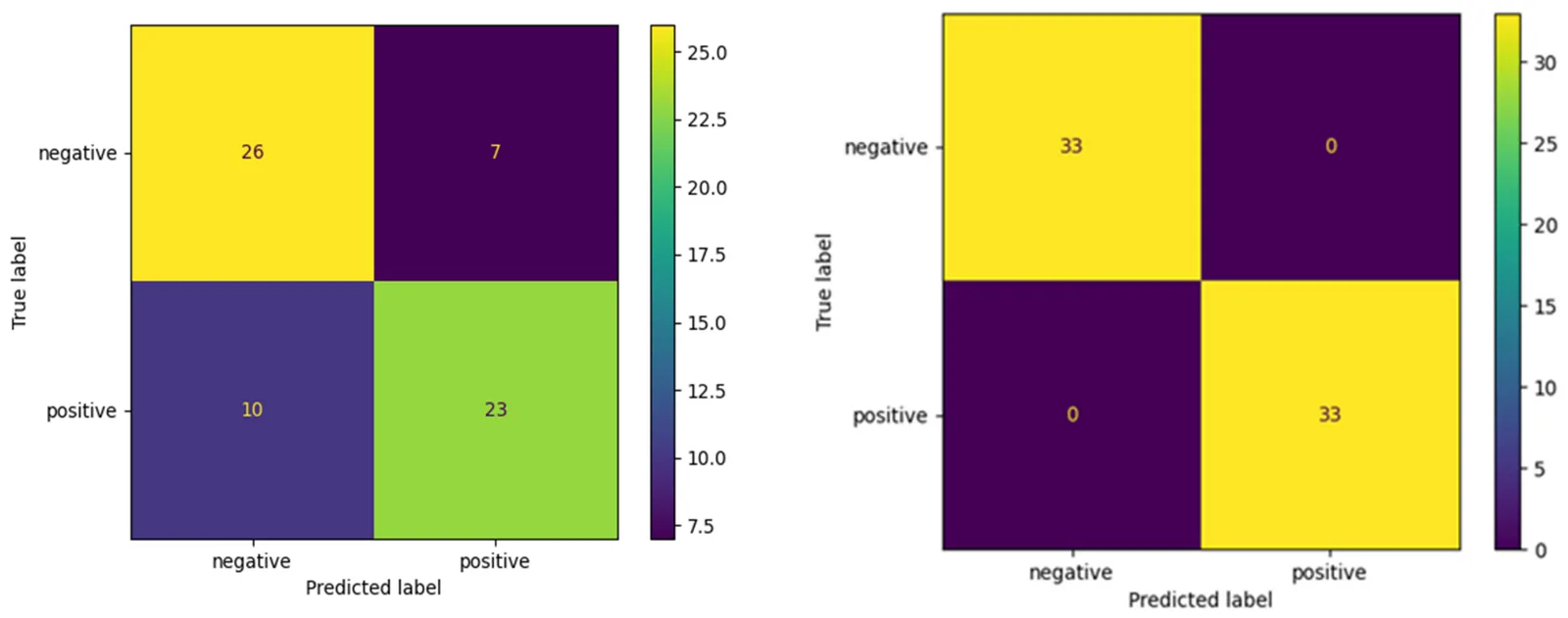
The confusion matrices for ResNet-50 and MobileNetV2.

Two models show exceptional performance: Xception achieved optimal classification (100% on all performance metrics, 95% CI [94.6%, 100%]) with the lowest loss (0.00048), indicating higher prediction certainty, while MobileNetV2 matched this optimal performance with 2.3x quicker training efficiency (10 m 12 s vs. 23 m 40 s). Both models had zero misclassifications (TP = 33, TN = 33, FP = 0, and FN = 0) out of 66 test patches. Although the point estimates for these two models (together with DenseNet201, InceptionV3, VGG-16, and VGG-19) reached 100%, to characterize the classification errors underlying the reported metrics, Figure 11 presents the confusion matrices for MobileNetV2 and ResNet-50 as representative cases spanning the observed performance range. MobileNetV2 achieved a fully diagonal matrix (33 true positives, 33 true negatives, and zero errors). ResNet-50 misclassified 17 of 66 patches, with a sensitivity of 69.70% (23/33) markedly lower than its specificity of 78.79% (26/33), indicating a tendency to underdetect *H. pylori*-positive cases rather than overdiagnose negative ones. The lower bound of the 95% CI (94.6%) indicates that a true accuracy as low as approximately 97% remains statistically compatible with the observed results, and this interval should be considered when interpreting the practical significance of these findings. ResNet-50, by contrast, showed both a markedly lower point estimate (74.24%) and a correspondingly wider confidence interval ([62%, 84.2%]), reflecting greater uncertainty in its performance estimate. The Vision Transformer achieved 96.97% accuracy (95% CI [89.5%, 99.6%]) on the test set.

To assess whether these differences were statistically robust, we conducted exact McNemar’s tests for comparisons against zero-error reference architectures, for which the discordant pair structure is fully determined by marginal error counts alone: ResNet50’s gap relative to the zero-error architectures was highly significant (*p* = 1.16 × 10^−10^), whereas the difference between the Vision Transformer and the zero-error architectures was not statistically significant (*p* = 0.125), indicating this gap may reflect sampling variability given the limited effective test sample size. Because six architectures achieved zero patch-level test errors across all 10 test patients (seven positive cases, three negative cases), their patient-level sensitivity, specificity, PPV, and NPV are, by construction, also 100%.

## 6. Discussion

In this study, we applied deep learning techniques for *H. pylori* classification utilizing transfer learning and Bayesian optimization for hyperparameter tuning. We selected eight different deep learning architectures, namely, MobileNetV2, DenseNet201, ResNet-50, InceptionV3, Xception, VGG-16, VGG-19, and ViT. These architectures performed excellently in the classification of the ImageNet datasets. This research aims to investigate the capacity of these models to extract useful features from the histopathology images of Egyptian patients. Despite using less data than some of the previous works, our models showed very good performance in discriminating between *H. pylori*-positive and negative cases. Furthermore, both MobileNetV2 and Xception models achieved 100% classification AUC-ROC and accuracy.

The use of Bayesian optimization for hyperparameter tuning represents a methodological advancement in our study. A comprehensive hyperparameter space was tested using a systematic approach, including the choice of optimizer, learning rate, batch size, and dropout, resulting in 50–80 different configurations per architecture. The optimal hyperparameters achieved for our final models played a pivotal role in enhancing the performance of our final models, with MobileNetV2 and Xception achieving perfect classification (see Table 8).

The current study reveals that deep learning models may achieve an impressive performance in distinguishing histopathological image classes. MobileNetV2 and Xception are two models that produced remarkable results. The results are noteworthy as both models show zero classification error on the test set (see Figure 10). However, given the modest size of the test set, the 95% confidence interval for this point estimate extends down to 94.6%, and this uncertainty should be kept in mind when interpreting the clinical significance of the reported performance. MobileNetV2 trains in a more efficient manner (10 m 12 s); Xception takes 23 m 40 s to train yet achieves perfect accuracy (see Table 8). This would be advantageous in practice, particularly with clinical cases that are resource-strapped. Nonetheless, the relatively small, single-institution size of the dataset is a limitation that may restrict generalizability to different clinical populations and imaging conditions. This is partially offset by patch-level extraction (432 patches from 100 WSIs), stratified splitting preserving stain and class balance, and the systematic Bayesian hyperparameter search across 50–80 configurations per architecture, which together reduce—though do not eliminate—the risk of overfitting to this particular data split. Future work should draw on more diverse multi-institutional data.

The single-institution origin of this dataset represents an important boundary on the generalizability of the reported results. Prior work in this domain has shown that model performance can degrade substantially when applied to out-of-distribution data. For instance, Parra-Medina et al. (2025) [17] reported a drop in overall accuracy from 97% to 78% upon external validation, driven primarily by reduced sensitivity on unfamiliar staining and imaging conditions. External validation of the top-performing architectures identified here (MobileNetV2, Xception) on independent datasets differing in scanning hardware, staining batches, and patient population is therefore essential before these models can be considered generalizable and constitutes a planned next phase of this research program.

The architectural comparison shows which deep learning techniques are best suited for histopathological image classification. ResNet-50 achieved a lower performance (74.24% accuracy) than other models, meaning not every architecture is equally suited to *H. pylori*. The performance gap for ResNet-50 (74.24%) is unlikely to stem from preprocessing or search space asymmetry, as all architectures shared an identical protocol. Its training curves (Figure 4) show persistent oscillation and a lower plateau than the other architectures, suggesting its residual connections required a longer fine-tuning schedule or more conservative learning rate than optimal under our fixed frozen backbone regime, a hypothesis we identify for future ablation testing rather than confirm here. As shown in Table 8, architectures such as DenseNet201, InceptionV3, VGG-16, and VGG-19 managed to accomplish perfect test accuracy (100%). The Vision Transformer, which relies on self-attention rather than convolutions, achieved an accuracy of 96.97%, which is competitive but slightly lower than the best convolutional network architectures. It seems that for the specific classification of patches sized 512 × 512 pixels, convolutional architectures preserve some benefits. Certainly, convolutional architectures come with built-in inductive biases for hierarchies and textures, making them particularly well suited for histopathology.

It was beneficial to train the models using transfer learning with ImageNet weights, as it allows the models to use low-level and mid-level visual features learned from millions of natural images.

Our models are difficult to compare directly with previous studies, as these differ not only in model architecture and dataset size, but also in staining procedures, patch dimensions, patient-level segregation practices, and validation strategies, any of which can materially affect reported accuracy and AUC independent of the underlying model quality. As such, although our results show outstanding performance, the comparison in Table 9 should be interpreted as contextual reference illustrating the broader feasibility of deep learning across diverse *H. pylori* histopathology settings, rather than as a rigorous head-to-head benchmark; readers are encouraged to weigh the methodological columns in Table 9 (dataset size, validation strategy, and patch size) alongside the performance figures when interpreting relative standing across studies.

The practical clinical implications of this study are even further than the direct diagnostic assignment. The development of computationally efficient models with a high degree of accuracy, like MobileNetV2, offers prospects of point-of-care implementation in environments with low computational capabilities. Our highest sensitivity rates of 100% with our best models are especially clinically relevant because this may indicate potential use as a screening test that can indicate suspicious cases to be examined further by experts (this could lower the false-negative rate of diagnosing *H. pylori* when there are few colonies or morphologically weak colonies in the microscopic results). Table 9. Comparison with previous studies of *H. pylori.*

In addition, systematic comparison of eight different architectures with rigorous Bayesian optimization helps future researchers and practitioners choose the right models for similar tasks. The fact that relatively light architectures can perform as well as or better than a large model has important practical implications for deployment feasibility as well as computational cost in a clinical setting.

Despite these remarkable results, our research has some limitations. The dataset used has only 432 images from one Egyptian hospital. Although this is the first curated Egyptian *H. pylori* dataset for deep learning, its relatively small size and single-center nature may limit the generalizability of the proposed models. While the reported metrics reflect a single training run per architecture at its Bayesian-optimized configuration, the frozen-backbone design in which only the lightweight classification head is trained atop fixed ImageNet-pretrained features inherently limits the scope for run-to-run variability relative to full end-to-end training. The reported 95% confidence intervals (Clopper-Pearson exact method) account for the finite test-set size, the primary source of estimation uncertainty in this setting. Nonetheless, we acknowledge that repeating training across multiple random seeds for the classification head, with mean ± SD reporting, would offer an additional layer of verification and is recommended as part of future multicenter validation of this framework. Furthermore, additional validation on larger, multicenter datasets is needed to assess the robustness and generalizability of the proposed approach and to support its scalability for broader clinical deployment. Future research directions include (1) external validation of the top-performing models (MobileNetV2, Xception) on independent multicenter Egyptian and regional datasets, encompassing variation in scanners, staining protocols, and patient populations, to rigorously assess generalizability prior to clinical deployment; (2) incorporating explainability techniques such as Grad-CAM to gain trust from Egyptian pathologists; (3) larger patient cohorts to narrow reported confidence intervals and enable statistically robust architecture comparisons; (4) exploration of hybrid AI models that combine the strengths of ViTs and CNNs; (5) exploring the possibility of developing an internet of things/AI platform that can allow pathologists to upload histopathological images and obtain results in real time; and (6) working with the Egyptian Ministry of Health to create AI guidelines for adoption, ensuring quality and access.

## 7. Conclusions

To overcome these problems, we have developed a comprehensive deep learning approach for automated *H. pylori* detection. To our knowledge, this is the first study that has applied artificial intelligence and deep learning techniques for *H. pylori* detection in Egypt and the Middle East region. We evaluated eight deep learning models on 432 histopathological images. We used Bayesian optimization to discover the best hyperparameters for each architecture.

The results were remarkable. Two models, namely, MobileNetV2 and Xception, hit all the metrics at 100% accuracy, sensitivity, specificity, precision, and F1-score. The main advantage of MobileNetV2 is that it trains more than twice as fast as Xception. DenseNet201, InceptionV3, VGG-16, and VGG-19 all achieved 100% accuracy on the test set. Vision Transformer achieved 96.97%. The exception was ResNet-50, which only reached 74.24% accuracy, despite optimization, suggesting it is less well suited to *H. pylori* classification using our experimental settings.

This study demonstrates that optimized deep learning models can achieve outstanding performance in *H. pylori* classification. To the best of our knowledge, this is the first application of AI for *H. pylori* detection in Egypt, demonstrating the feasibility of adapting state-of-the-art deep learning approaches to Egyptian histopathology data. MobileNetV2’s high patch-level accuracy and computational efficiency make it a promising candidate for future deployment in Egyptian labs with limited computing power, contingent on external multicenter validation, prospective clinical evaluation, and appropriate regulatory review prior to any clinical use.

## Figures and Tables

**Table 2:** (**a**) Distribution of histopathological patches across training, validation, and test sets. (**b**) The data augmentation techniques used and their ranges.

**(a)**			
**Subset**	***H. pylori*** **-positive**	***H. pylori*** **-negative**	**Total**
Training	150	150	300
Validation	33	33	66
Testing	33	33	66
Overall	216	216	432
**(b)**			
**Technique**		**Range**	
Rotation range		0–15°	
Width shift range		10%	
Height shift range		10%	
Shear range		10%	
Zoom range		10%	
Horizontal flip range		True	
Vertical flip range		True	
Fill mode		Nearest	

**Table 3:** Hyperparameter search space for Bayesian optimization.

Hyperparameter	Search Space/Range
Optimizer	[“Adam”, “RMSprop”, “SGD”, “Adagrad”, “Adadelta”, “Adamax”, “Nadam”]
Learning rate	[1 × 10^−2^, 1 × 10^−3^, 1 × 10^−4^, 1 × 10^−5^]
Batch size	[8, 16, 32, 64]
Apply dropout	[True, False]
Dropout (if true)	[0.1, 0.2, 0.3, 0.4, 0.5]

**Table 4:** The different performance metrics utilized.

Metric	Definition	Equation
Accuracy (ACC)	It is the number of correct predictions divided by the total number of predictions.	$ACC=\frac{TP+TN}{TP+TN+FP+FN}$
Area under the ROC curve (AUC)	The average of each point in the ROC curve and also reflects the separability degree	-
Precision	The percentage between the number of true positive results and the total number of positive results.	$P=\frac{TP}{TP+FP}$
Recall or sensitivity	The percentage between the number of true positive results and the total relevant samples.	$Recall=\frac{TP}{TP+FN}$
F1 score	The harmonic mean of precision and recall. Its limits are [0, 1]. A higher F1 score indicates a higher model’s performance.	$F1=\frac{2TP}{2TP+FP+FN}$
Specificity	The ability to detect a true negative value and identify people who did not have a specific condition or a disease.	$S=\frac{TN}{FP+TN}$

**Table 5:** Experimental setup for all models.

Configuration	Specifications
Model	MobileNetV2, DenseNet201, ResNet-50, InceptionV3, Xception, VGG-16, VGG-19, Vision Transformer (ViT)
Libraries	Keras, Tensorflow, matplotlib, OS, Numpy, OpenCV, Scikit-learn
Class mode	Categorical
Input layer	512 × 512 × 3
Accuracy	Confusion matrix
Activation function	Softmax
Loss	Categorical crossentropy
Learning rate	Optimized for each architecture
Batch size	Optimized for each architecture
Optimizer	Optimized for each architecture
Epochs	50
Output layer	2

**Table 6:** Representative subset of the optimal hyperparameter configurations evaluated for each model (selected from the full set of 50–80 trials searched per architecture).

Model	Optimizer	Learning Rate	Batch Size	Apply Dropout (True/False)	Dropout
MobileNetV2	RMSprop	0.01	8	False	0.0
MobileNetV2	RMSprop	0.001	16	True	0.4
MobileNetV2	Adamax	0.001	8	False	0.0
MobileNetV2	SGD	0.01	32	True	0.1
MobileNetV2	Adam	0.01	16	False	0.0
MobileNetV2	Adam	0.01	8	False	0.0
MobileNetV2	RMSprop	0.001	32	True	0.5
MobileNetV2	Nadam	0.001	32	True	0.5
DenseNet201	SGD	0.001	32	True	0.5
DenseNet201	Adam	0.001	32	True	0.5
DenseNet201	SGD	0.001	32	False	0.0
DenseNet201	Adam	0.001	32	False	0.0
DenseNet201	Adamax	0.01	64	False	0.0
DenseNet201	RMSprop	0.001	32	True	0.4
ResNet50	Adamax	0.0001	64	False	0.0
ResNet50	Adamax	0.001	32	False	0.0
ResNet50	Adamax	0.01	64	True	0.1
ResNet50	SGD	0.001	64	True	0.1
ResNet50	Adamax	0. 001	32	True	0.5
ResNet50	Adagrad	0.001	32	True	0.5
InceptionV3	Nadam	0.001	16	False	0.0
InceptionV3	Adam	0.001	64	False	0.0
InceptionV3	Nadam	0.001	16	False	0.0
InceptionV3	Adamax	0.001	64	False	0.2
InceptionV3	Adam	0.0001	16	True	0.4
InceptionV3	Adam	0.01	64	True	0.4
Xception	Adam	0.01	8	False	0.0
Xception	Adamax	0.001	32	True	0.3
Xception	Nadam	0.0001	64	True	0.4
Xception	Adamax	0.001	64	True	0.4
Xception	Adamax	0.001	32	True	0.3
Xception	Nadam	0.001	32	True	0.5
Xception	Adamax	0.001	32	True	0.5
VGG16	RMSprop	0.001	32	True	0.4
VGG16	RMSprop	0.001	32	True	0.2
VGG16	RMSprop	0.001	32	True	0.5
VGG16	RMSprop	0.001	32	True	0.3
VGG16	RMSprop	0.01	8	False	0.0
VGG16	Adam	0.001	32	True	0.5
VGG16	Nadam	0.001	32	True	0.5
VGG16	Adam	0.0001	8	False	0.0
VGG16	Adamax	0.001	64	True	0.5
VGG19	Adam	0.001	16	True	0.5
VGG19	Adamax	0.001	32	True	0.5
VGG19	RMSprop	0.001	32	True	0.5
VGG19	Adagrad	0.01	32	True	0.5
VGG19	Adagrad	0.01	32	False	0.0
VGG19	Adam	0.001	64	True	0.3
VGG19	Nadam	0.01	64	False	0.0
VGG19	Adam	0.01	64	False	0.0
ViT	Adam	0.0001	32	False	0.0
ViT	RMSprop	0.0001	16	True	0.1
ViT	RMSprop	0.0001	8	False	0.0
ViT	Adam	0.0001	16	False	0.0

**Table 7:** The best configuration of hyperparameters for all models.

Model	Optimizer	Learning Rate	Batch Size	Apply Dropout (True/False)	Dropout	Training Accuracy	Validation Accuracy	Testing Accuracy	Time	Epoch
MobileNetV2	RMSprop	0.01	8	False	0.0	0.9967	1.0	1.0	10 min 12 s	24
MobileNetV2	RMSprop	0.001	16	True	0.4	0.9933	1.0	1.0	16 min 56 s	41
MobileNetV2	Adamax	0.001	8	False	0.0	0.9933	1.0	1.0	17 min 3 s	41
MobileNetV2	SGD	0.01	32	True	0.1	0.9800	1.0	1.0	19 min 6 s	50
MobileNetV2	Adam	0.01	16	False	0.0	0.9967	1.0	1.0	12 min 46 s	30
MobileNetV2	Adam	0.01	8	False	0.0	0.9933	1.0	1.0	15 min 21 s	37
MobileNetV2	RMSprop	0.001	32	True	0.5	0.9900	1.0	1.0	20 min 6 s	50
MobileNetV2	Nadam	0.001	32	True	0.5	0.9900	1.0	1.0	20 min 48 s	47
DenseNet201	SGD	0.001	32	True	0.5	0.9667	0.9848	0.9848	28 min 58 s	50
DenseNet201	Adam	0.001	32	True	0.5	0.9967	1.0	1.0	29 min 30 s	50
DenseNet201	SGD	0.001	32	False	0.0	0.9733	0.9848	0. 9697	32 min 24 s	50
DenseNet201	Adam	0.001	32	False	0.0	0.9767	1.0	0.9848	21 min 24 s	36
DenseNet201	Adamax	0.01	64	False	0.0	0.9867	1.0	0.9849	18 min 17 s	27
DenseNet201	RMSprop	0.001	32	True	0.4	0.9833	1.0	0.9848	23 min 44 s	41
ResNet50	Adamax	0.0001	64	False	0.5	0.7699	0.8485	0.7273	26 min 25 s	50
ResNet50	Adamax	0.001	32	False	0.0	0.7933	0.8333	0.7424	24 min 21 s	50
ResNet50	Adamax	0.01	64	True	0.1	0.7733	0.8333	0.7424	25 min 39 s	50
ResNet50	SGD	0.001	64	True	0.1	0.7567	0.7879	0.7273	24 min 59 s	50
ResNet50	Adamax	0. 001	32	True	0.5	0.7767	0.8333	0.7424	27 min 11 s	50
ResNet50	Adagrad	0.001	32	True	0.5	0.7799	0.8333	0.7273	27 min 24 s	50
InceptionV3	Nadam	0.001	16	False	0.0	1.0	1.0	1.0	23 min 25 s	48
InceptionV3	Adam	0.001	64	False	0.0	0.9967	1.0	1.0	25 min 46 s	50
InceptionV3	Nadam	0.001	16	False	0.0	0.9967	1.0	1.0	20 min 18 s	42
InceptionV3	Adamax	0.001	64	False	0.0	0.9867	1.0	1.0	22 min 46 s	50
InceptionV3	Adam	0.0001	16	True	0.4	.0.9867	1.0	1.0	25 min 30 s	50
InceptionV3	Adam	0.01	64	True	0.4	0.9900	1.0	0.9848	25 min 26 s	50
Xception	Adam	0.01	8	False	0.0	1.0	1.0	1.0	27 min 3 s	50
Xception	Adamax	0.001	32	True	0.3	0.9967	1.0	1.0	27 min 1 s	50
Xception	Nadam	0.0001	64	True	0.4	0.9833	0.9848	1.0	34 min 22 s	50
Xception	Adamax	0.001	64	True	0.4	0.9900	0.9848	1.0	24 min 58 s	46
Xception	Nadam	0.001	32	True	0.5	1.0	1.0	1.0	23 min 40 s	45
Xception	Adamax	0.001	32	True	0.5	0.9733	0.9848	0.9848	23 min 47 s	39
VGG16	RMSprop	0.001	32	True	0.4	0.9967	1.0	0.9848	33 min 33 s	50
VGG16	RMSprop	0.001	32	True	0.2	0.9933	1.0	1.0	24 min 29 s	50
VGG16	RMSprop	0.001	32	True	0.5	0.9967	1.0	0.9848	24 min 15 s	50
VGG16	RMSprop	0.001	32	True	0.3	0.9933	1.0	0.9697	25 min 57 s	50
VGG16	RMSprop	0.01	8	False	0.1	0.9900	1.0	0.9848	24 min 38 s	49
VGG16	Adam	0.001	32	True	0.5	0.9967	1.0	0.9848	46 min 25 s	50
VGG16	Nadam	0.001	32	True	0.5	0.9967	1.0	0.9848	24 min 11 s	50
VGG16	Adam	0.0001	8	False	0.3	0.9967	1.0	0.9848	23 min 10 s	50
VGG16	Adamax	0.001	8	True	0.5	0.9900	0.9848	0.9848	23 min 39 s	50
VGG19	Adam	0.001	16	True	0.5	0.9933	1.0	0.9848	27 min 48 s	50
VGG19	Adamax	0.001	32	True	0.5	0.9800	0.9848	0.9848	26 min 22 s	50
VGG19	RMSprop	0.001	16	True	0.5	0.9933	1.0	0.9848	25 min 53 s	50
VGG19	Adagrad	0.01	32	True	0.5	0.9633	0.9848	0.9696	25 min 39 s	50
VGG19	Adagrad	0.01	32	False	0.1	0.9633	0.9848	0.9848	25 min 53 s	50
VGG19	Adam	0.001	32	True	0.3	.0.9966	1.0	0.98484	28 min 2 s	50
VGG19	Nadam	0.01	32	False	0.3	0.9933	1.0	0.98484	24 min 13 s	46
VGG19	Adam	0.01	32	False	0.3	0.9933	1.0	1.0	24 min 40 s	50
ViT	Adam	0.0001	32	False	0.0	1.0	1.0	0.9697	22 min 51 s	50
ViT	RMSprop	0.0001	16	True	0.1	0.9896	0.9844	0.9375	22 min 6 s	41
ViT	RMSprop	0.0001	8	False	0	0.9966	1.0	0.9531	19 min 40 s	38
ViT	Adam	0.0001	16	False	0	0.96875	0.984375	0.953125	18 min 34 s	33

**Table 8:** The best configuration of hyperparameters for each model (95% confidence intervals for test-set performance metrics).

Model	Optimizer	Learning Rate	Batch Size	Apply Dropout (True/False)	Dropout	Training Accuracy	Validation Accuracy	Testing Accuracy (95% CI)	Sensitivity (95% CI)	Specificity (95% CI)	Time	Epoch
MobileNetV2	RMSprop	0.01	8	False	0.0	0.9967	1.0	1.0 [0.946, 1.0]	1.0 [0.894, 1.0]	1.0 [0.894, 1.0]	10 min 12 s	24
DenseNet201	Adam	0.001	32	True	0.5	0.9967	1.0	1.0 [0.946, 1.0]	1.0 [0.894, 1.0]	1.0 [0.894, 1.0]	29 min 30 s	50
ResNet50	Adamax	0.001	32	False	0.1	0.7933	0.8333	0.7424 [0.62, 0.842]	0.697 [0.513, 0.844]	0.7879 [0.611, 0.91]	24 min 21 s	50
InceptionV3	Nadam	0.001	16	False	0.0	1.0	1.0	1.0 [0.946, 1.0]	1.0 [0.894, 1.0]	1.0 [0.894, 1.0]	23 min 25 s	48
Xception	Nadam	0.001	32	True	0.5	1.0	1.0	1.0 [0.946, 1.0]	1.0 [0.894, 1.0]	1.0 [0.894, 1.0]	23 min 40 s	45
VGG16	RMSprop	0.001	32	True	0.2	0.9933	1.0	1.0 [0.946, 1.0]	1.0 [0.894, 1.0]	1.0 [0.894, 1.0]	24 min 29 s	50
VGG19	Adam	0.01	32	False	0.3	0.9933	1.0	1.0 [0.946, 1.0]	1.0 [0.894, 1.0]	1.0 [0.894, 1.0]	24 min 40 s	50
ViT	Adam	0.0001	32	False	0.0	1.0	1.0	0.9697 [0.895, 0.996]	0.9697 [0.842, 0.999]	0.9697 [0.842, 0.999]	22 min 51 s	50

**Table 9:** Comparison with previous studies of *H. pylori*.

Authors & Year (Ref.)	Dataset	Validation Stain	Dataset Size (WSI)	Deep Learning Architecture	AUC	Additional Preprocessing	Hyperparameter Optimization Method	Geographic Region	Validation Strategy	Execution Time (Training)
Proposed approach (this study)	Institutional	H&E, Giemsa	100	MobileNetV2 DenseNet201 ResNet-50 InceptionV3 Xception VGG-16 VGG-19 ViT	1.0 0.9999 0.8457 0.9999 1.0 0.9999 0.9999 0.9934	Image resizing (512 × 512), patch extraction (1024 × 1024), data augmentation (rotation, zoom, width and height shift, shear, horizontal and vertical flipping, and fill mode)	Bayesian optimization (seven types of optimizers, four learning rates, four batch sizes, and various dropout values)	Egypt	Hold-out validation with train/validation/test split (~70%/~15%/~15%) respectively	Yes
Parra-Medina et al., 2025 [17]	Institutional	H&E	100	InceptionV3 VGG16 Resnet50 BoostedNet AutoKeras	1.0 1.0 1.0 0.92 0.92	Quality filtering: exclusion of patches with > 60% background or lacking visible tissue	Manual tuning + AutoML	Colombia	Hold-out (train–test split (80% training/20% testing))	NO
Krishna et al., 2024 [28]	Public dataset	H&E, Giemsa	19	BoostedNet: CNN + XGBoost	0.990	Image resizing (256 × 256), data augmentation (rotation, zoom, shear, flip), Gaussian filtering	Manual tuning	India	Hold-Out (train–test split (70% training/30% testing))	NO
Ibrahim et al., 2024 [16]	Institutional	H&E	204	DenseNet-201 EfficientNet-b0 MobileNet-V2 ResNet-101 Xception	0.9383 0.9383 0.9486 0.9417 0.9176	N/A	Manual tuning	Turkey	Cross-Validation (Five-fold cross-validation)	NO
Lin et al., 2023 [29]	Institutional	H&E	1075	ESCNN + Logistic Regression	0.9739	N/A	Manual tuning	Taiwan	Hold-Out (Train–test split)	NO
Franklin et al., 2022 [27]	Institutional	H&E	187	HALO-AI software (fully convolutional VGG network)	N/A	Data augmentation (rotations variations in hue, saturation, contrast, and brightness)	Not specified	United States	Hold-Out (Train–test split (60% training/40% testing))	NO
Goncalves et al., 2022 [20]	Public dataset	H&E	19	VGG16 InceptionV3 ResNet50	0.998 0.994 0.994	Splitting WSIs into patches (256 × 256 pixels), conversion to grayscale, median masking for noise reduction, removal of patches with > 50% white background, data augmentation (rotation, flip, and zoom)	Manual tuning	Brazil	Hold-out (train–test split (70% training, 15% validation, 15% testing))	NO
Klein et al., 2020 [10]	Institutional	H&E, Giemsa	627	Compact VGG-style deep neural network	0.950 0.902 0.810	Active learning (iterative labeling of high-scoring *H. pylori* crops); RGB to HSV conversion; Otsu’s thresholding for tissue detection	Manual tuning	Germany	Independent hold-out	NO
Zhou et al., 2020 [18]	Institutional	H&E	303	CNN ensemble of 3 DenseNet-121 (top) and 3 ResNet-18 (bottom)	0.965	Laplacian filtering, data augmentation through horizontal inversion	Manual tuning	United States	Hold-out validation with train/validation/test split (47/126/130) WSI, respectively	NO

## Data Availability

The original contributions presented in this study are included in the article. Further inquiries can be directed to the corresponding authors.
